# Phagocytosis by Thrombocytes is a Conserved Innate Immune Mechanism in Lower Vertebrates

**DOI:** 10.3389/fimmu.2014.00445

**Published:** 2014-09-16

**Authors:** Takahiro Nagasawa, Chihaya Nakayasu, Aja M. Rieger, Daniel R. Barreda, Tomonori Somamoto, Miki Nakao

**Affiliations:** ^1^Department of Bioscience and Biotechnology, Graduate School of Bioresource and Bioenvironmental Sciences, Kyushu University, Fukuoka, Japan; ^2^National Research Institute of Aquaculture, Fisheries Research Agency, Minami-Ise, Japan; ^3^Department of Biological Sciences, University of Alberta, Edmonton, AB, Canada

**Keywords:** thrombocyte, phagocytosis, comparative immunology, platelet, innate immunity, fish immunity

## Abstract

Thrombocytes, nucleated hemostatic blood cells of non-mammalian vertebrates, are regarded as the functional equivalent of anucleated mammalian platelets. Additional immune functions, including phagocytosis, have also been suggested for thrombocytes, but no conclusive molecular or cellular experimental evidence for their potential ingestion and clearance of infiltrating microbes has been provided till date. In the present study, we demonstrate the active phagocytic ability of thrombocytes in lower vertebrates using teleost fishes and amphibian models. *Ex vivo*, common carp thrombocytes were able to ingest live bacteria as well as latex beads (0.5–3 μm in diameter) and kill the bacteria. *In vivo*, we found that thrombocytes represented nearly half of the phagocyte population in the common carp total peripheral blood leukocyte pool. Phagocytosis efficiency was further enhanced by serum opsonization. Particle internalization led to phagolysosome fusion and killing of internalized bacteria, pointing to a robust ability for microbe elimination. We find that this potent phagocytic activity is shared across teleost (*Paralichthys olivaceus*) and amphibian (*Xenopus laevis*) models examined, implying its conservation throughout the lower vertebrate lineage. Our results provide novel insights into the dual nature of thrombocytes in the immune and homeostatic response and further provide a deeper understanding of the potential immune function of mammalian platelets based on the conserved and vestigial functions.

## Introduction

Phagocytosis is a crucial mechanism in innate immune defense against microbes and in homeostatic clearance of apoptotic cells and their debris (1). Among the various blood cells, neutrophils and macrophages/monocytes in the myeloid lineage have long been recognized as professional or classic phagocytes in a wide range of vertebrate species, including fish and mammals (2). Phagocytes also play a key role in triggering the adaptive immune response by presenting foreign antigens to helper T-cells through MHC class II molecules (1, 3).

In recent years, B-lymphocytes of teleost fishes, amphibians, and reptiles have been shown to be active phagocytes (4, 5), suggesting an ancestral function of B cells in innate immunity. Furthermore, phagocytosis by non-myeloid leukocytes such as B-1 cells and gamma/delta-T-cells has also been reported for mammals (6–9), renovating our understanding on the phagocytic cell population and its significance in innate and adaptive immunity.

Hemostasis is another mechanism against microbial infection through blood vessel repair, wherein mammalian platelets and non-mammalian thrombocytes play a triggering role in blood coagulation (10). Thrombocytes of non-mammalian vertebrates are nucleated leukocytes and are considered to be the functional counterparts of mammalian platelets (11). Platelets are small cell fragments released from megakaryocytes, playing an essential role in triggering hemostasis (12). In addition to homeostatic functions, the possible roles of thrombocytes in immunity, such as phagocytosis, have also been raised in various species, including teleost fishes and birds (13–17). Mechanistic insights into the phagocytic ability of these non-mammalian thrombocytes and their impact on pathogen clearance, however, have long been controversial because of contradictory lines of experimental evidence (6, 18–20). For example, morphological observations of thrombocytes showed putative phagocytosis of microbes (13–17), but cytoenzymatic analysis showed inconsistent result to support this idea, particularly in lower vertebrates such as teleosts (20–23). Moreover, the apparent target internalization is questioned because thrombocytes possess an extensive canalicular system open to the extracellular surface, which is a network of interconnected channels of the cell membrane to broaden their cell surfaces and release various intracellular components (19, 24), making it possible to trap particles in a passive manner (19, 25, 26).

In mammals, platelets express various immune-related molecules such as Toll-like receptors and Fc-receptors for antigen recognition (27–32). Expression of proinflammatory proteins such as IL-1β and CD40L also depicts potential immunomodulatory functions of platelets (33–35); however, their ability to internalize microbes also remains controversial with contrariety data (25, 26, 36–39).

In this study, we used a combined cell biology and molecular approach, employing animal models and cell culture techniques, to assess the phagocytic capacity of teleost and amphibian thrombocytes and their capacity to mediate downstream intracellular anti-microbial killing responses. Our functional assessment of the phagocytic responses of thrombocytes complements recent molecular analyses, which showed that thrombocytes express genes involved in antigen presentation and the inflammatory response (40, 41). The functional significance and evolutionary implications of the phagocytic thrombocytes are discussed in relation with the immune functions of mammalian platelets.

## Materials and Methods

### Animals

Common carp (*Cyprinus carpio*, 50–100 g) were maintained in our laboratory at 25°C and fed with commercial pellets. Ginbuna crucian carp (*Carassius auratus langsdorfii*, approximately 50 g) were hatched and maintained in our laboratory at 25°C. Goldfish (*Carassius auratus auratus*, approximately 70 g) were maintained at the University of Alberta (Edmonton, AB, Canada). Japanese flounder (*Paralichthys olivaceus*, approximately 200 g) were maintained in the Fisheries Research Institute, Oita Prefectural Agriculture, Forestry, and Fisheries Research Center (Oita, Japan)*. Xenopus laevis* (approximately 80 g) were purchased from a commercial farm.

All the animal experiments except in goldfish were performed in accordance with the guidelines of the Animal Experiments Committee at Kyushu University. The experiment in goldfish was performed in accordance with the guidelines of the Canadian Council on Animal Care and the University of Alberta Animal Care and Use Committee.

### Isolation of fish and frog leukocytes

Peripheral blood samples were drawn from caudal veins of the common carp anesthetized in 50 ppm 2-methylquinoline (Kanto Chemical Co., Tokyo, Japan) into heparinized syringes, diluted with RPMI-1640 (Nissui Pharmaceutical Co., Tokyo, Japan), and overlaid onto Percoll adjusted to 1.08 g/ml (BD Biosciences, San Jose, CA, USA), followed by centrifugation at 500 × *g* for 30 min at 4°C to isolate peripheral blood leukocytes (PBLs). PBLs at the top of the Percoll layer were washed twice with RPMI-1640 by centrifugation at 500 × *g* for 10 min at 4°C and adjusted at 1 × 10^7^ cells/ml with RPMI-1640. PBLs of ginbuna, goldfish, and flounder were essentially collected in the same manner. Peripheral blood samples of *Xenopus* were obtained from the heart after anesthetization with diethylether (Kanto Chemical) and separated as above.

### Cell staining, separation, and flow cytometry

Common carp PBLs were incubated with an HB8 (24) monoclonal antibody (mAb) for 30 min on ice. After washing twice with RPMI-1640, samples were incubated with MACS (magnetic activated cell sorting) microbeads coupled to the goat anti-mouse IgG antibody (Miltenyi Biotec, GmbH, Germany). After washing twice, samples were resuspended in RPMI-1640 containing 2 mM EDTA and 10% fetal bovine serum (FBS) and loaded on a mini MACS column (Miltenyi) to purify antibody-positive cells. For the assessment of purity, collected samples were stained with fluorescein isothiocyanate (FITC)-conjugated anti-mouse IgG goat antibody (Sigma-Aldrich Co., St. Louis, MO, USA) for 30 min on ice, washed twice, resuspended in PBS, and analyzed on a flow cytometer (EPICS XL; Beckman Coulter, Brea, CA, USA). Single cells were gated by forward scatter and side scatter as described in Figure S1C in Supplementary Material. For the detection of each cell population, crucian carp and goldfish PBLs were stained with GB10 (anti-thrombocyte), 6D1 (anti-CD4), 2C3 (anti-CD8α), and B12 [anti-immunoglobulin M (IgM)] mAbs (42). Flounder PBLs were stained with JFW10 (anti-flounder thrombocyte) and JFW20 (anti-flounder IgM) mAbs (43). *X. laevis* PBLs were stained with T12 (anti-*X. laevis* thrombocyte) mAb provided by Dr. Takashi Kato (Yuta Tanizaki, Takako Ishida-Iwata, Miyako Obuchi-Shimoji, Takashi Kato, Cellular characterization of thrombocytes in *X. laevis* with specific monoclonal antibodies, manuscript in submission).

### RT-PCR

Total RNA was purified from purified thrombocytes (1 × 10^6^ cells, purity >99%) and lysed using 1 ml of ISOGEN reagent (Wako Chemical Co., Osaka, Japan) according to the manufacturer’s instruction. cDNA was synthesized with Moloney murine leukemia virus (M-MLV) reverse transcriptase (Life Technologies) for 60 min at 37°C. The cDNA corresponding to 0.5 μg of RNA served as a template for PCR using Taq-polymerase (Sigma), which was performed under the following conditions: 24–35 cycles of 15 s at 95°C, 15 s at 52–56°C, and 30 s at 72°C, depending on the primer pairs. PCR products were analyzed by 2% agarose gel electrophoresis and stained with ethidium bromide. Primer sequences and actual cycle numbers are shown in Table S1 in Supplementary Material.

### Phagocytosis assay

Peripheral blood leukocytes (1 × 10^7^ cells/ml) were incubated with fluorescent latex beads (Fluoresbrite Yellow Green Microspheres; Polysciences, Warrington, PA, USA) at a cell to bead ratio of 1:5 in RPMI-1640 containing 5% FBS for 3 h at 25°C in 100 μl. The cells were incubated with anti-thrombocyte mAb for 30 min at 4°C, washed twice, and stained with phycoerythrin (PE)-conjugated anti-mouse IgG goat antibody (Sigma) for 30 min at 4°C, followed by flow cytometry and fluorescence microscopy (Eclipse; Nikon, Tokyo, Japan). Flounder and *X. laevis* PBLs were incubated at 20°C.

### Transmission electron microscope analysis

Purified thrombocytes (1 × 10^6^ cells) were pelleted and prefixed with 2.5% glutaraldehyde and 2% paraformaldehyde in phosphate buffer (pH 7.4) for 60 min on ice. After washing twice, pellets were stained with 2% tannic acid in PBS for 4 h at 4°C. Pellets were post-fixed with 1% osmic acid in PBS for 60 min at 4°C, dehydrated with ethanol and propylene oxide, and embedded in Epon 812 (Shell Chemicals, Rotterdam, Netherlands). Ultrathin sections were cut and stained with uranyl acetate and lead acetate and observed under a transmission electron microscope (TEM) (Hitachi H-7000; Hitachi, Ltd., Tokyo, Japan).

### Phagocytosis of bacteria

Opsonization efficiency analysis of bacteria with immunoglobulin or complement FITC-conjugated *Escherichia coli* were incubated with 5% non-immunized common carp serum in PBS in the presence of 2 mM Mg^2+^ and Ca^2+^ for 30 min at 25°C. As controls, bacteria were incubated with heat-inactivated (for 20 min at 50°C) non-immunized antiserum or carp antiserum immunized by fixed *E. coli* containing 10 mM EDTA under the same conditions. After washing with PBS three times, the treated bacteria were adjusted to OD_600 nm_ = 0.5 and incubated with common carp PBLs (1 × 10^7^ cells/ml) for 3 h at 25°C. PBLs were stained with HB8 mAb and PE-conjugated anti-mouse IgG goat antibody as mentioned above, and analyzed by flow cytometry and fluorescent microscopy. Fluorescence of non-internalized bacteria was quenched with 0.2% Trypan Blue (Wako).

### Assessment of *in vivo* phagocytosis

Common carp (three individuals with approximate body weight of 20 g) anesthetized with 50 ppm 2-methylquinoline were received an intravenous injection of 1 × 10^7^ fluorescent beads (1 μm in diameter) in 50 μl of PBS. After 3 h, the fish were euthanized by 10-fold overdose of 2-methylquinoline and bled, followed by isolation of PBLs as described above. Spleen and kidney were taken and minced through a stainless steel mesh soaked in RPMI-1640. Thus, obtained cell suspensions were filtered through 50 μm nylon mesh (Beckman) and overlaid onto the Percoll for removal of erythrocytes by centrifugation, stained with HB8 mAb and PE-conjugated anti-mouse IgG goat antibody as mentioned above, and analyzed by flow cytometry and fluorescent microscopy.

### Phagolysosome fusion assay

Goldfish PBLs stained with GB10 mAb and PE-conjugated anti-mouse IgG antibody were incubated with 2.5% FITC–dextran (10,000 MW, anionic, lysine-fixable, Life Technologies) in D-MEM (Life Technologies) for 45 min at 25°C. After washing and preincubating in D-MEM for 45 min at 25°C, the cells were incubated with non-fluorescent 3-μm beads (Polysciences) at the ratio of 1:5 in D-MEM for 3 h at 25°C. The cells were then fixed with 1% formaldehyde in PBS and analyzed on ImageStream (Amnis, Seattle, WA, USA). For LysoTracker analysis, common carp PBLs were incubated with non-fluorescent 3-μm beads for 3 h at 25°C, stained with HB8 mAb and FITC-conjugated anti-mouse IgG goat antibody, and stained with 1 μM of LysoTracker^®^ Red (Life Technologies) in PBS for 15 min at 4°C, followed by fluorescent microscopy. For assessment of phagolysosome fusion against bacteria, common carp thrombocytes incubated with FITC-conjugated *E. coli* were purified with HB8 mAb as mentioned above, stained with LysoTracker Red, and analyzed in the same manner.

### Intracellular killing assay

Common carp PBLs were incubated with green fluorescent protein (GFP)-expressing live *E. coli* (DH5α, OD_620 nm_ = 0.5) in RPMI-1640 containing 5% FBS for 3 h at 25°C, and thrombocytes were purified as described above. The cells were then incubated with 100 μg/ml of gentamycin (Sigma) in RPMI-1640 for 2 h at 25°C to kill non-internalized bacteria and incubated in RPMI-1640 for 0, 3, and 6 h, followed by hypotonic lysis with deionized water. The lysates were diluted, spread onto LB plates, and incubated at 37°C for 24 h. Bacterial colonies were then counted, and the results are presented as percentages of the number of colonies at 0 h of incubation.

## Results

### Gene expression in common carp thrombocytes

**Figure 1:**
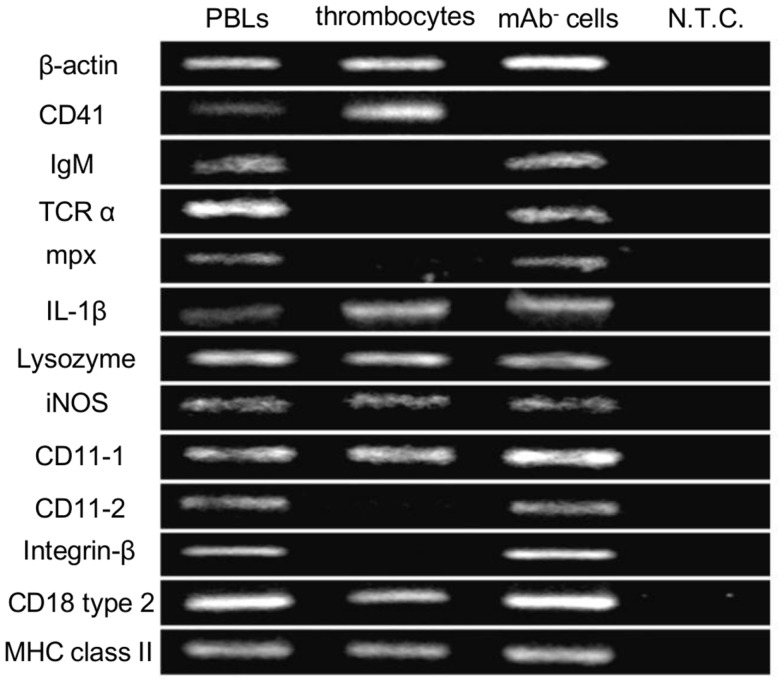
**Gene expression profiles of common carp thrombocytes and other leukocytes are shown**. Expression of molecules on total PBLs, MACS-purified HB8 mAb^+^ thrombocytes, and HB8 mAb^-^ cells. N.T.C., no template control; β-actin, internal control; IgM, immunoglobulin M; TCRα, T-cell receptor α chain; mpx, myeloperoxidase; IL-1β, interleukin-1β; iNOS, inducible nitric oxide synthase; MHC class II, major histocompatibility complex class II. Data are representative of five independent experiments.

As an initial step to characterize non-mammalian thrombocytes, we analyzed the expression patterns of several immune-relevant genes in the common carp isolates. We purified the common carp thrombocytes using an mAb that was previously shown to be specific (HB8 mAb) coupled with a MACS^®^ magnetic separation system (24). With this method, we obtained a highly purified thrombocyte fraction (purity >99%, Figures S1A,B in Supplementary Material). The purified HB8^+^ thrombocytes express the CD41 transcript (also referred to as glycoprotein IIb or integrin α IIb), a specific marker of platelets and thrombocytes in various species [(44, 45); Figure 1]. CD41 expression was not detected in the HB8-negative fraction, in contrast to the expression of other typical leukocyte markers such as IgM for B cells, T-cell receptor α (TCRα) chain for T cells, and myeloperoxidase (mpx) for neutrophils. Notably, the purified thrombocytes expressed IL-1β, a typical proinflammatory cytokine, as well as several lysozymes and inducible nitric oxide synthase (iNOS), all involved in bactericidal activities (46). We also examined the expression of CD11/CD18, the putative common carp orthologs of the mammalian complement receptor 3 (CR3) subunits (47). Interestingly, only one of the two isoforms of each CD11 and CD18 (CD11-1 and CD18 type 2) are detected in common carp thrombocytes, whereas other leukocyte fractions express both isoforms, suggesting selective expression of CD11/CD18 isoforms in different leukocyte lineages. These data suggested that thrombocytes have a complete complement receptor 3 complex and may be able to recognize complement-opsonized antigens. Finally, MHC class II, a central contributor to antigen presentation of internalized particles, was also detected in the thrombocyte fraction. Taken together, the above data suggest that common carp thrombocytes recognize opsonized antigens, have bactericidal abilities, play a role in the induction of inflammatory cascades, and may also bridge innate and adaptive immunity by antigen presentation.

### Phagocytic thrombocytes and parameters influencing their activity

**Figure 2:**
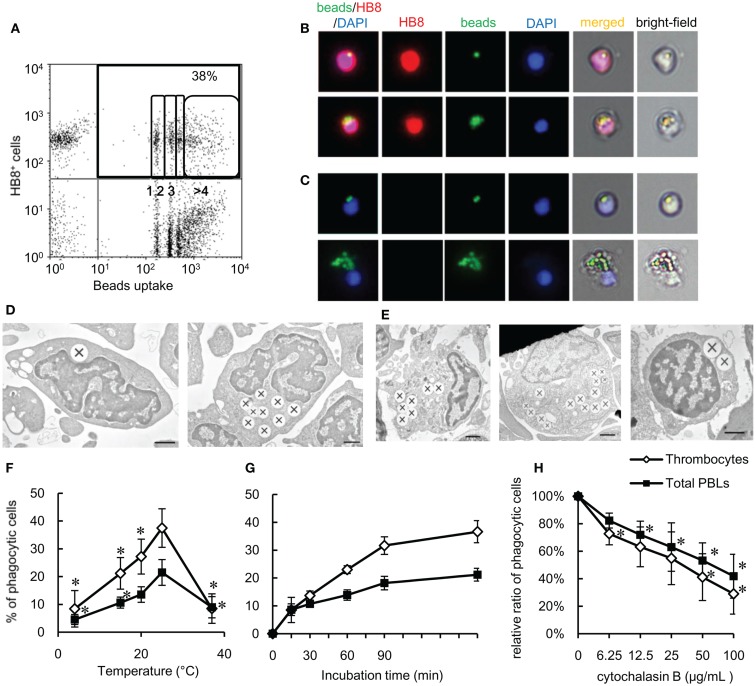
**Phagocytosis by common carp thrombocytes and other leukocytes is shown**. **(A)** Flow cytometry of common carp PBLs incubated with fluorescent beads measuring 1 μm in diameter for 3 h at 25°C. The percentage indicates the rate of phagocytic cells in HB8^+^ thrombocytes. The numbers (1, 2, 3, and >4) indicate the number of ingested beads within each gated cell analyzed by fluorescence intensity on flow cytometry. **(B,C)** Fluorescent microscopy of phagocytic common carp thrombocytes with one [**(B)**, upper] and several [**(B)**, lower] latex beads measuring 1 μm in diameter and HB8 mAb^-^ phagocytes with two [**(C)**, upper] and several [**(C)**, lower] beads. Red, HB8 mAb specific for common carp thrombocytes; green, FITC-beads; blue, nuclei stained with DAPI. Original magnification, ×400. Data are representative of eight independent experiments. **(D,E)** Representative transmission electron micrographs of phagocytic thrombocytes **(D)** and other phagocytes **(E)** incubated with beads measuring 1 μm in diameter. Ingested beads are indicated as X. [**(E)**, left] Granulocyte-like cell, [**(E)**, center] monocyte-like cell, [**(E)**, right] lymphocyte-like cell. Data are representative of three independent experiments. Original magnification, ×10,000. **(F–H)** Parameters influencing the phagocytosis of common carp thrombocytes and other leukocytes. The relationship of the phagocytic ability of common carp thrombocytes and total PBLs with temperature (4, 15, 20, 25, and 37°C) **(F)**, incubation time **(G)**, and concentration of cytochalasin B **(H)**. Data are expressed as the average of at least three independent experiments shown as mean ± SD. The asterisks (*) indicate significant differences from the mean of the same population at 25°C (**F**) or that with no cytochalasin B (**H**), as analyzed by Student’s *t*-test (*P* < 0.05).

To functionally test our above hypothesis, we assayed the phagocytic capacity of common carp thrombocytes. To this end, common carp PBLs were incubated with fluorescent 1-μm latex beads. Flow cytometric analysis first revealed that a large percentage (38 ± 6%; Figure 2A) of HB8^+^ thrombocytes interacted with beads, with a considerable number being associated with four or more beads. Under a fluorescent microscope, most of the beads interacted by thrombocytes seemed to be ingested, not only surface-bound (Figure 2B). To determine if these particles were truly internalized or not, we analyzed the interacting thrombocytes by TEM and found that the majority of particles were ingested by thrombocytes (Figure 2D). The number of cells with ingested beads as observed by fluorescent microscopic observations was similar to that measured by flow cytometry (Figure S1D in Supplementary Material), suggesting that the majority of particles detected by flow cytometry were indeed internalized and not surface-bound. Interestingly, the majority of bead-associated thrombocytes had changed from the typical spindle or oval shapes to round or adherent forms (Figures 2B,C; Figure S1B in Supplementary Material), suggesting that phagocytic interactions induced morphological changes.

As previously shown (24, 48), we confirmed by TEM ultrastructural observations that the thrombocytes had a heterochromatin-rich nucleus and several small vacuoles (Figure 2D). Internalized beads were tightly clustered in the cytoplasm, similar to beads internalized by other HB8^-^ phagocytes (Figure 2E). The phagocytic thrombocytes with several beads have distortions in their nuclear morphology (Figure 2D, right).

We then tested the effects of temperature and time on the efficiency of phagocytosis by carp thrombocytes. Phagocytic activity was enhanced when the temperature is increased from 4 to 25°C, with a peak phagocytic activity at 25°C (Figure 2F) and a dramatic decrease at 37°C. Phagocytosis also increased over time, with a maximum of 37 ± 4% found after 180 min (Figure 2G). Importantly, the phagocytic activity of thrombocytes was inhibited by cytochalasin B in a dose-dependent manner, indicating that bead uptake requires active actin polymerization, as observed in other classical phagocytes (Figure 2H). These data support the hypothesis that thrombocytes are true phagocytes that can ingest foreign particles in the same manner as that of professional phagocytes.

### Comparison of phagocytic ability in leukocytes

**Figure 3:**
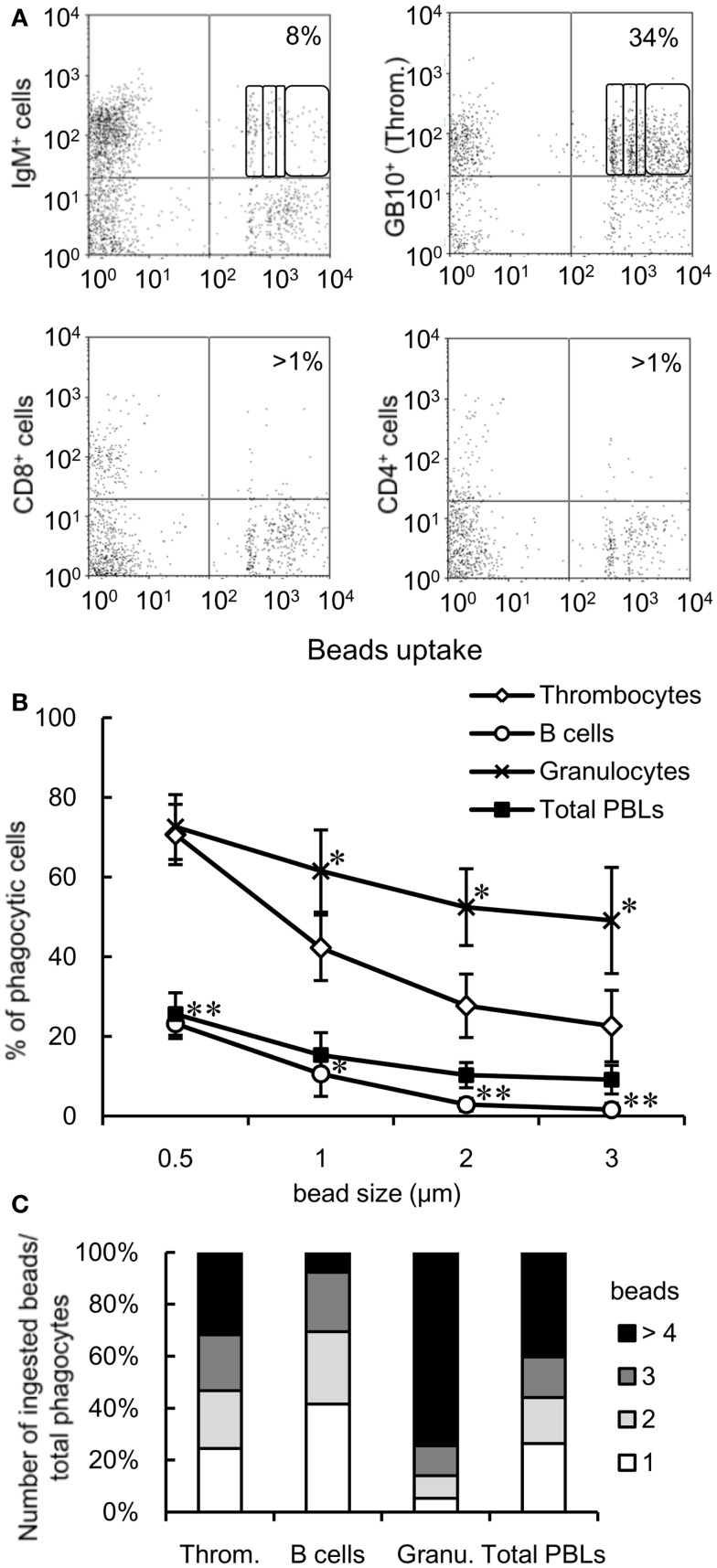
**Comparison of the phagocytic activities of each cell subpopulation is shown**. **(A)** Flow cytometry of mAb-stained crucian carp PBLs incubated with fluorescent latex beads measuring 1 μm in diameter for 3 h at 25°C. The percentage indicates the rate of phagocytic cells in each mAb^+^ cell population. The numbers (1, 2, 3, and >4) indicate the number of ingested beads within each gated cell analyzed by fluorescence intensity on flow cytometry. Data are representative of three independent experiments. Throm., GB10 mAb^+^ cells; CD4^+^ cells, 6D1 mAb^+^ cells; CD8^+^ cells, 2C3 mAb^+^ cells; IgM^+^ cells, B12 mAb^+^ cells. **(B)** Percentage of phagocytic cells in each cell population of common carp PBLs incubated with each size of beads for 3 h at 25°C analyzed by flow cytometry. Data are expressed as the average of four independent experiments, shown as mean ± SD. The asterisks (*) denote significant differences from the mean of thrombocytes incubated with the same beads, as analyzed by Student’s *t*-test (*P < 0.05, **P < 0.01). **(C)** Number of ingested beads measuring 1 μm in diameter in the phagocytic cells of each cell population as analyzed by fluorescence intensity on flow cytometry. Throm., HB8 mAb^+^ thrombocytes; Granu., granulocytes. Data are expressed as the average of four independent experiments.

To determine the relative contribution of thrombocytes to the phagocytic elimination of foreign particles, we compared the phagocytic capacity of thrombocytes with that of other leukocyte populations, particularly phagocytic lymphocytes, which have received a lot attention in recent years (4–9). We assessed the phagocytic abilities of thrombocytes and each lymphocyte subpopulation in the close common carp relative, ginbuna crucian carp (*Carassius auratus langsdorfii*), in which mAbs specific for IgM (B12), CD4 (6D1), and CD8α (2C3) and specific lymphocyte-type mAbs are available (42). In line with previous studies, we found a significant percentage of phagocytic IgM^+^ B cells (8 ± 3%, Figure 3A). Interestingly, this percentage is largely below the phagocytic GB10 mAb^+^ thrombocyte (34 ± 6%) percentage, and, functionally, the thrombocytes can internalize more beads per cell compared with B cells. Neither CD4^+^ nor CD8^+^ PBL fractions showed notable phagocytic activities.

We tested the responses to granulocytes (as a classic example of the professional phagocyte pool) and assessed the impact of particle size on phagocytosis. Granulocytes were defined by their forward and side scatter properties using flow cytometry (Figure S1C in Supplementary Material). When PBLs were incubated with different sizes of beads (0.5, 1, 2, or 3 μm in diameter), the number of phagocytic cells decreased in a size-dependent manner (Figure 3B). Over 20% thrombocytes were capable of ingesting larger beads (2 and 3 μm in diameter), while <3% B cells (stained with CI-14 mAb specific for common carp IgM) ingested these beads. On comparing the phagocytic index of each cell population using 1-μm beads, approximately 30% phagocytic thrombocytes were capable of ingesting four or more beads, while <10% phagocytic B cells were able to ingest that amount (Figure 3C). These results suggested that thrombocytes have potent phagocytic capacity and may play a greater role in phagocytosis compared with the recently described phagocytic B cells in common carp.

### Bacterial phagocytosis by thrombocytes

**Figure 4:**
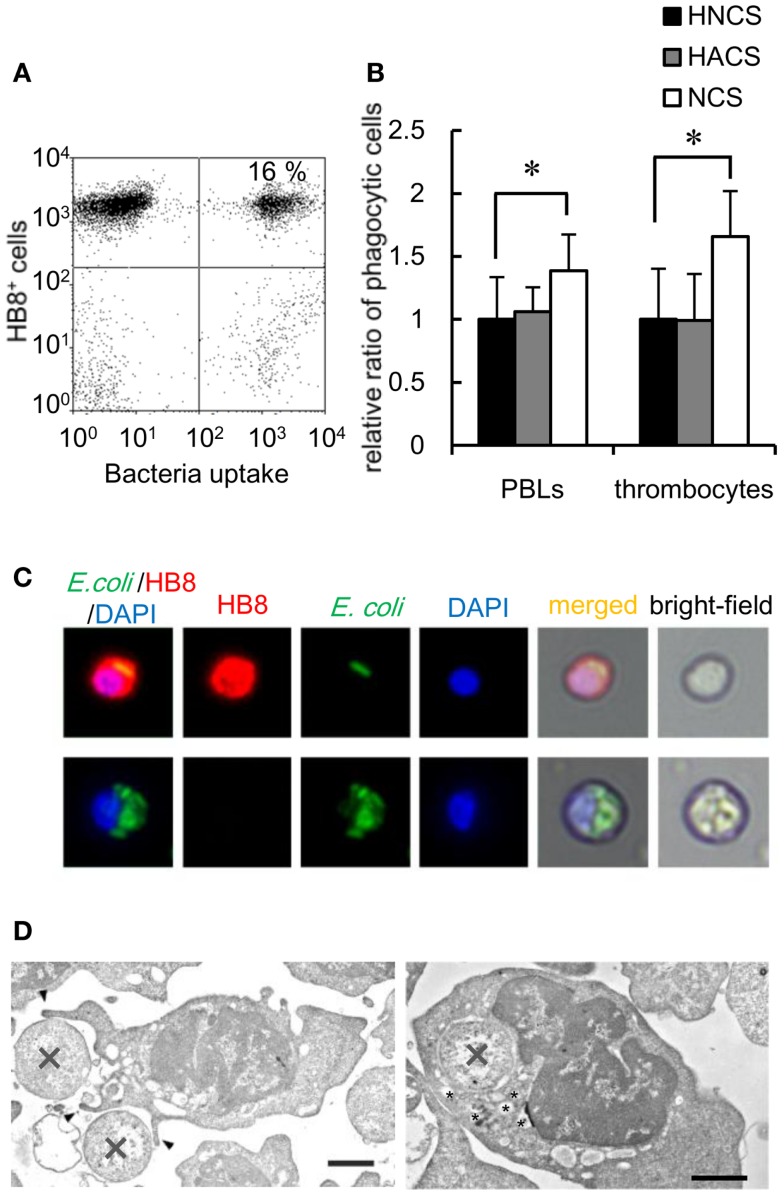
**Bacterial phagocytosis by thrombocytes and other leukocytes is shown**. **(A)** Flow cytometry of common carp PBLs incubated with FITC-conjugated *E. coli* for 3 h at 25°C. The number indicates the percentage of phagocytic cells in HB8^+^ thrombocytes. **(B)** Quantification of the effects of oposonization by incubation with heat-inactivated normal carp serum (HNCS), heat-inactivated anti-*E. coli* carp serum (HACS), and normal carp serum (NCS). Data are expressed as the average of five independent experiments, shown as mean ± SD. The asterisks (*) indicate significant differences from the control, as analyzed by Student’s *t*-test (*P* < 0.05). **(C)** Phagocytic common carp thrombocytes (upper) and mAb^-^ phagocyte (lower)-ingested bacteria; red, HB8 mAb; green, bacteria; blue, nuclei stained with DAPI. Original magnification, ×400. **(D)** Representative transmission electron micrographs of phagocytic thrombocytes in the process of ingesting bacteria (X) via the extension of pseudopods (arrowhead; left) and with ingested bacteria (right). Small vesicles surrounding the internalized bacteria are indicated by asterisks (*). Bar, 1 μm. Original magnification, ×10,000.

To determine the capacity of thrombocytes to phagocytose natural pathogens, common carp PBLs were incubated with formaldehyde-fixed *E. coli* conjugated with FITC. The results of flow cytometric analysis showed that common carp thrombocytes could phagocytose bacteria (16 ± 4%; Figure 4A). The effect of opsonization was tested using FITC-*E. coli* preincubated with common carp serum as a source of active complement. Following opsonization, we found a significant increase in the number of phagocytic thrombocytes (166 ± 36%) compared with that of control bacteria treated with heat-inactivated common carp serum in the presence of EDTA (Figure 4B). This suggests that common carp thrombocytes express complement receptors that contribute to the efficiency of phagocytic uptake, as recognized for other professional phagocytes. On the other hand, treatment with heat-inactivated antiserum showed no opsonizing effects on common carp PBLs under our experimental condition.

Fluorescent microscopy and TEM analysis also demonstrated the ingestion of bacteria by thrombocytes (Figures 4C,D). TEM analysis showed thrombocytes interacting with bacteria by the extension of pseudopods (Figure 4D, left). Tannic acid (26), which stains extracellular surfaces (Figure 4D, left; Figures S2A,B in Supplementary Material), did not stain ingested bacteria (Figure 4D, right; Figure S2B in Supplementary Material), indicating that bacteria were truly internalized by these thrombocytes. Interestingly, the phagocytic thrombocytes had many small vacuoles around the internalized bacteria (Figure 4D, right; Figure S2B in Supplementary Material), suggesting that bacterial internalization triggers the production of lysosomal vacuoles containing enzymes and other reactive molecules for the digestion of particles within the phagosomes.

### *In vivo* study of phagocytic thrombocytes

**Figure 5:**
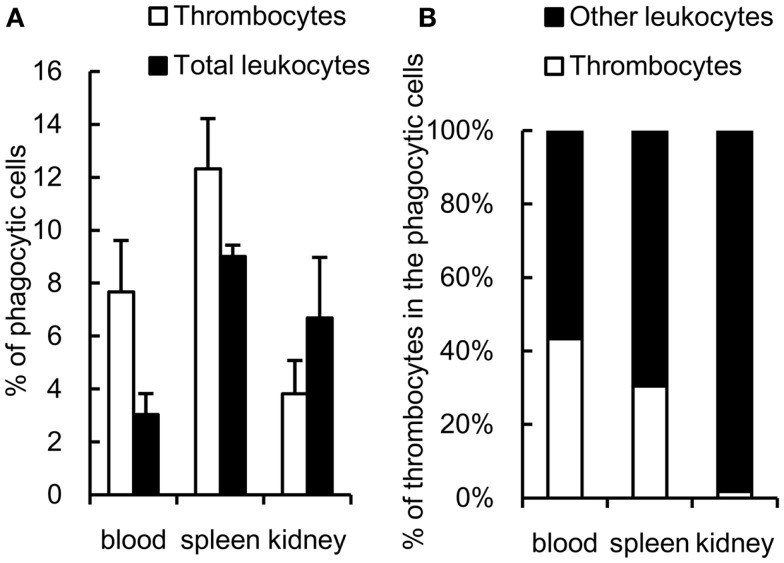
***In vivo* study of common carp phagocytic thrombocytes and other leukocytes 3 h after intravenous injection**. **(A)** Percentage of phagocytic cells in HB8 mAb^+^ thrombocytes and total leukocytes in each tissue. **(B)** Percentage of thrombocytes and other HB8 mAb^-^ leukocytes in phagocytic populations in each tissue. Data are expressed as the average of three independent experiments, shown as mean ± SD.

The phagocytic activity of thrombocytes was further assessed *in vivo* by injecting fluorescent 1-μm beads into the caudal vein. At 3 h after ingestion, 7 ± 2% of peripheral blood thrombocytes showed effective phagocytosis of beads (Figure 5A). The phagocytic thrombocytes accounted for nearly half the total phagocytic cells in the blood (Figure 5B). Interestingly, phagocytic thrombocytes were also detected in the spleen and kidney, suggesting that thrombocytes may play an important role in the clearance of circulating antigens by subsequent transport to these lymphoid tissues.

### Phagolysosome fusion and intracellular killing of thrombocytes

**Figure 6:**
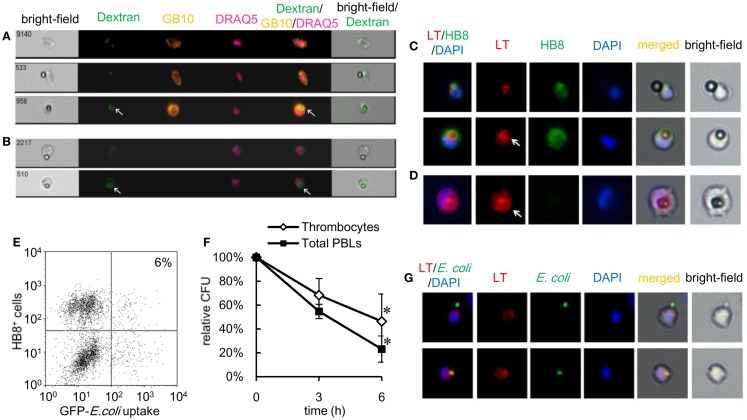
**Phagolysosome fusion and intracellular killing by thrombocytes and other leukocytes are shown**. **(A)** Goldfish phagocytic thrombocytes stained with fluorescent dextran and incubated with non-fluorescent latex beads measuring 3 μm in diameter for 3 h at 25°C. Thrombocyte alone (upper), with bound (middle), or ingested (lower) beads. **(B)** GB10 mAb^-^ goldfish phagocytes with bound beads (upper) and ingested beads (lower) and a phagolysosome ring (arrow). A phagolysosome ring is observed around the ingested particle (arrow). Dextran, FITC–dextran; GB10, mAb for goldfish thrombocytes; DRAQ5, nuclei. **(C,D)** Common carp phagocytic thrombocytes stained with LysoTracker Red to visualize acidic organelles. **(C)** Thrombocytes with bound (upper) or ingested (lower) non-fluorescent latex beads measuring 3 μm in diameter. **(D)** Phagocytic mAb^-^ leukocyte with an ingested bead (arrow). The phagolysosome ring as detected by acidic probe staining. LT (red), acidic organelles visualized by LysoTracker Red; HB8 (green), HB8 mAb specific for common carp thrombocytes; DAPI (blue), nuclei stained with DAPI. Original magnification, ×400. Data are representative of three independent experiments. **(E,F)** Intracellular bactericidal activity of thrombocytes. **(E)** Flow cytometry of phagocytic common carp thrombocytes incubated with GFP-expressing live *E. coli* for 3 h at 25°C. The number indicates the percentage of phagocytic cells in HB8^+^ thrombocytes. Data are representative of three independent experiments. **(F)** Survival rate of internalized bacteria counted on Luria–Bertani agar plates. Data are expressed as the average of three independent experiments, shown as mean ± SD. The asterisks (*) indicate significant differences from the mean of 0 h, as analyzed by Student’s *t*-test (*P* < 0.05). **(G)** MACS-purified common carp thrombocytes with surface-bound (upper) or ingested (lower) GFP-expressing *E. coli*. Colocalization of lysosomal organelles is observed around ingested bacteria (arrowhead). LT, acidic organelles visualized by LysoTracker^®^ Red; *E. coli*, bacteria; DAPI, nuclei stained with DAPI. Original magnification, ×400. Data are representative of three independent experiments.

Activation of intracellular killing mechanisms is critical to the anti-microbial contribution of phagocytes. Pathogen internalization is followed by the fusion of phagosomes and lysosomes, exposing the internalized pathogen to anti-microbial proteins and reactive molecules. Therefore, we evaluated whether thrombocytes were armed with such a killing capacity by characterizing phagolysosome fusion following particle internalization. Using goldfish (*Carassius auratus auratus*) as another subspecies of crucian carp, PBLs were stained with fluorescent dextran, which accumulates in lysosomal organelles (49, 50). Goldfish PBLs stained with FITC–dextran were incubated with non-fluorescent 3-μm beads, tagged with GB10 mAb, and analyzed on an ImageStream^®^ multispectral image flow cytometer. When particles are unbound or surface-bound, no lysosomal staining is observed in association with the beads (Figure 6A, upper and middle). In contrast, internalized beads colocalized with an FITC–dextran phagolysosome ring that could be observed around each bead (Figure 6A, lower). Similar results are observed in mAb GB10-negative phagocytes (Figure 6B). To corroborate this observation, we also examined phagolysosome fusion in common carp thrombocytes using LysoTracker^®^ Red as an indicator of acidic organelles (51). Common carp PBLs were incubated with beads and stained with HB8 mAb and LysoTracker, followed by fluorescent microscopy. In agreement with the above results, common carp thrombocytes formed phagolysosome rings only when they ingested the beads (Figure 6C), as observed in other classical phagocytes (Figure 6D).

Anti-microbial functions of phagocytic thrombocytes were further assessed using a gentamicin protection assay, a common method used to test intracellular bactericidal activity (52), with live *E.coli*-GFP as the target. As shown in Figure 6E, common carp thrombocytes ingested the live bacteria, although the number of phagocytic cells was much lower (6 ± 2%) than that previously observed with fixed bacteria (Figure 6E). The survival rate of internalized bacteria decreased in a time-dependent manner in both thrombocytes and other phagocytes (Figure 6F). Furthermore, the LysoTracker-stained acidic organelles colocalized with the ingested bacteria (Figure 6G, lower), but not with surface-bound bacteria (Figure 6G, upper). Taken together, these data show that thrombocytes not only have the capacity to internalize bacteria but also possess potent bactericidal mechanisms to kill them.

### Phagocytic thrombocytes in neoteleosts and amphibians

**Figure 7:**
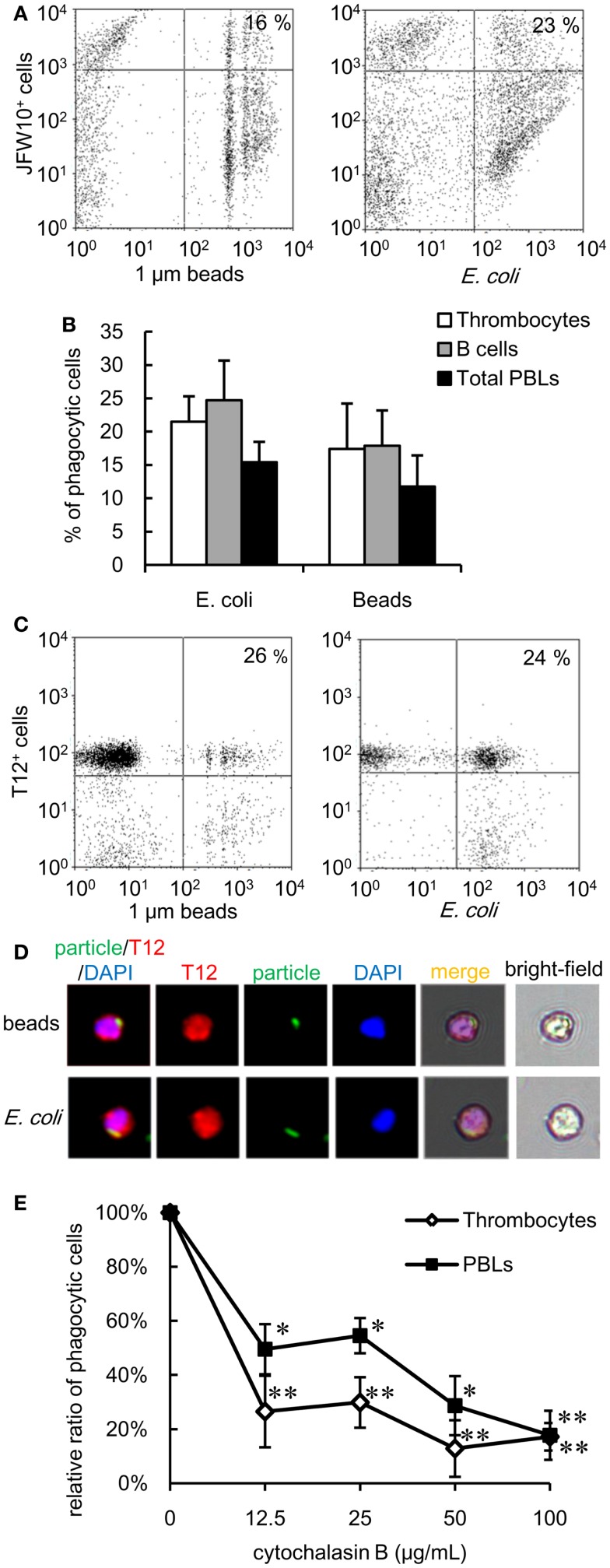
**Phagocytic thrombocytes in other vertebrates are shown**. **(A,B)** Phagocytosis by flounder thrombocytes and B cells. **(A)** Flow cytometry of flounder thrombocytes incubated with fluorescent latex beads measuring 1 μm in diameter and FITC-conjugated *E. coli* for 3 h at 20°C. The number indicates the percentage of phagocytic cells in JFW10^+^ thrombocytes. Data are representative of four independent experiments. **(B)** Percentage of phagocytic cells in each cell population of flounder. Data are expressed as the average of four independent experiments, shown as mean + SD. **(C,D)** Phagocytosis by *X. laevis* thrombocytes. **(C)** Flow cytometry of *X. laevis* thrombocytes incubated with fluorescent latex beads measuring 1 μm in diameter and FITC-conjugated *E. coli*. The number indicates the percentage of phagocytic cells in T12^+^ thrombocytes. **(D)** Phagocytic *X. laevis* thrombocytes with (upper) latex beads measuring 1 μm in diameter or (lower) FITC-conjugated *E. coli* for 3 h at 20°C. Red, T12 mAb specific for *X. laevis* thrombocytes; green, FITC-beads or *E. coli*; blue, nuclei stained with DAPI. Original magnification, ×400. Data are representative of three independent experiments. **(E)** Effect of the concentration of cytochalasin B to the phagocytosis by *X. laevis* thrombocytes and other leukocytes. Data are expressed as the average of three independent experiments, shown as mean ± SD. The asterisks (*) indicate significant differences from the control (no cytochalasin B), as analyzed by Student’s *t*-test (*P < 0.05, **P < 0.01).

The experiments described above highlight the phagocytic capacity of thrombocytes across several members of the teleost fish group. To further explore the phylogeny of thrombocyte phagocytic function, we compared the phagocytic capacity of Japanese flounder (*Paralichthys olivaceus*), a teleost species taxonomically distant from the cyprinids, and *X. laevis* as an amphibian model. Both models benefited from the availability of anti-thrombocyte mAbs (43). A flow cytometric assay showed that 17 ± 6 and 21 ± 4% of flounder thrombocytes ingested latex beads and bacteria, respectively (Figure 7A). Therefore, the phagocytic ability of thrombocytes is a general feature of teleost fish. In addition, the phagocytic activity of IgM^+^ B cells was assessed in parallel using an mAb specific for flounder IgM (43). In flounder, the phagocytosis capacity of B cells (latex beads, 18 ± 5%; *E. coli*, 24 ± 6%) was comparable with that of thrombocytes (Figure 7B). In a similar flow, cytometric analysis using *X. laevis* PBLs and an anti-*X. laevis* thrombocyte mAb (T12), active phagocytosis (beads: 26 ± 4%, *E. coli*: 24 ± 4%) was observed (Figures 7C,D). Similar to the fish thrombocytes, the phagocytosis by *X. laevis* thrombocytes was inhibited by cytochalasin B (Figure 7E). The above findings indicate that the phagocytic activity of thrombocytes is a fundamental feature conserved among lower vertebrates.

## Discussion

Accumulating lines of evidence have urged that bony fish is a promising animal model to pursue immunological and hematological research not only in phylogenetic context but also in ontogenetic and functional points of view. This has greatly been accelerated by *in vivo* studies using transgenic and mutant zebrafish (53, 54), in addition to several aquaculture species, such as rainbow trout, carp, and catfish, allowing functional studies at cellular and protein levels.

The current study shows that thrombocytes of lower vertebrates are armed with full phagocytic functions, in addition to their previously described ability for the production of cytokines that control immune responses. The overall characteristics of the phagocytosis by thrombocytes are fairly comparable with those of classical phagocytes such as macrophages and neutrophils, proposing its functional significance in the innate immune defense of lower vertebrates.

Important features of the phagocytosis by thrombocytes include the following: (1) ability to ingest particulate antigens measuring up to 3 μm in diameter as well as fixed and live bacteria in a manner dependent on actin polymerization, both *in vitro* and *in vivo*, (2) enhancement of the ingestion by opsonization with complement, and (3) formation of phagolysosomes, leading to a bactericidal consequence. We believe that these lines of functional evidence point to thrombocytes as bona fide phagocytes in lower vertebrates.

The relative contribution of the phagocytic thrombocytes to the total phagocytic capacity among the leukocyte pool warrants further consideration. While the ingesting capacity of each thrombocyte is lower than that of macrophages or neutrophils, thrombocytes are often the second most dominant blood cells after erythrocytes in the circulating blood of fish (nearly half the total phagocytes in blood as observed in the present study (43, 48, 55, 56), occasionally representing more than half of the total PBL population (55, 56). Therefore, thrombocytes may play a significant role in the uptake of particulate antigens in the blood circulation. It is interesting to note that phagocytic thrombocytes were also detected in the spleen and kidney, suggesting a potential role of thrombocytes in the transport of circulating bacterial antigens to lymphoid tissues, where these may trigger the adaptive immune response.

It will be interesting to compare the capacity of thrombocytes to present extrinsic antigens with that of B cells, macrophages, and dendritic cells in order to understand the relative contribution of each population to T-cell activation. This presents opportunities for increasing our current understanding of the evolution of cellular networks in innate pathogen recognition, immunomodulatory intercellular communication, and adaptive immune responses.

Opsonization of the target particle with fish serum contributes significantly to the efficiency of the phagocytosis by carp thrombocytes; however, the mechanism mediating the opsonizing effect remains to be clarified. It seems likely that a mammalian CR3-like receptor is responsible, at least in part, for the opsonized ingestion, on the basis of the detection of mRNA encoding CD18 and CD11b/CD11c-like integrin subunits in carp thrombocytes. Only CD11-1 and CD18-2 isotypes were detected in the thrombocyte (among the two duplicated isotypes identified in the common carp), indicating thrombocyte-specific expression of the CR3-subunit isotypes. The observation that heat-inactivation of serum prior to incubation with the target almost totally abrogated this opsonic effect also suggests complement-mediated opsonization.

The detection of IL-1β and MHC class II transcripts in thrombocytes suggests a potential role in inflammation and antigen presentation, in agreement with their expression in trout and chicken thrombocytes (40, 41). In addition, avian thrombocytes express various cytokines in response to stimulation with various TLR ligands (41), indicating that thrombocytes act as immunological sensors in circulating blood and may mediate immune responses against pathogens. A comprehensive expression analysis of a panel of cytokines would provide a more concrete picture of the immunomodulatory function of thrombocytes. In this context, it is intriguing to note that a very recent study on mammalian platelet stimulate transformation of monocytes into epithelioid-like multinucleated giant foam cells, suggesting its involvement in tuberculosis pathogenesis (57), giving a rise of possibility that platelets/thrombocytes-driven leukocyte differentiation. Immunomodulatory function and fate of phagocytic thrombocyte are now very interesting study topics to understand comprehensive role of thrombocytes both in anti-microbial defense by phagocytosis and in homeostasis by efferocytosis.

Our study highlights the conservation of the phagocytic capacity of thrombocytes across teleost fish and *X. laevis*, an amphibian model. Several lines of evidence are consistent with further conservation in birds (13, 17). From an evolutionary perspective, the present findings suggest strong conservation of the phagocytic function of thrombocytes throughout the vertebrate lineage. In this context, it is interesting that platelets responsible for mammalian hemostasis also express a wide variety of immunomodulatory cytokines, including IL-1β, CXCL4, and RANTES (32, 33, 58, 59). It has been reported that platelets can modulate adaptive immune responses via CD40L, which induces dendritic cell maturation, class switching of B cells, and CD8^+^ T-cell activation (12, 34, 35).

The present findings also provide insights into the evolution of hematopoietic cell lineages. Classically, platelets/thrombocytes and erythrocytes (erythroid cells) have been grouped into the same lineage as myeloid cells. In fact, their common progenitors (common myeloid/erythroid progenitor; CMEP) have been identified in the bone marrow and fetal liver of mice (60, 61). However, recent studies have proposed new lineage models, particularly the myeloid-based model, based on novel clonal assays (62). According to this model, phagocytic myeloid cells resemble the prototypic cells of blood cells, and the progenitors of each lineage possess the potential to differentiate into phagocytic cells. In fact, myeloid-like behaviors such as phagocytosis were observed on more immune cell types than previously thought, particularly in lower vertebrates (4–9). Phagocytosis is probably an innate or original function of cells of erythroid lineage in ancestral vertebrates and may represent a vestigial feature of platelets in higher vertebrates.

## Author Contributions

Takahiro Nagasawa, Tomonori Somamoto, and Miki Nakao designed the work. Takahiro Nagasawa performed the experiments. Chihaya Nakayasu performed the TEM analysis. Aja M. Rieger and Daniel R. Barreda contributed the analytic tools. Takahiro Nagasawa wrote the paper and Tomonori Somamoto and Miki Nakao supervised the experiments and co-wrote the manuscript.

## Conflict of Interest Statement

The authors declare that the research was conducted in the absence of any commercial or financial relationships that could be construed as a potential conflict of interest.

## Supplementary Material

The Supplementary Material for this article can be found online at http://www.frontiersin.org/Journal/10.3389/fimmu.2014.00445/abstract
