# Supplementary material for: Phagocytosis by Thrombocytes is a Conserved Innate Immune Mechanism in Lower Vertebrates
Source: Front Immunol. 2014 Sep 16;5:445. doi: 10.3389/fimmu.2014.00445 (PMC4165319; doi:10.3389/fimmu.2014.00445)
Supplement: Supplementary file 1 [file Presentation1.PDF]

Supplemental material

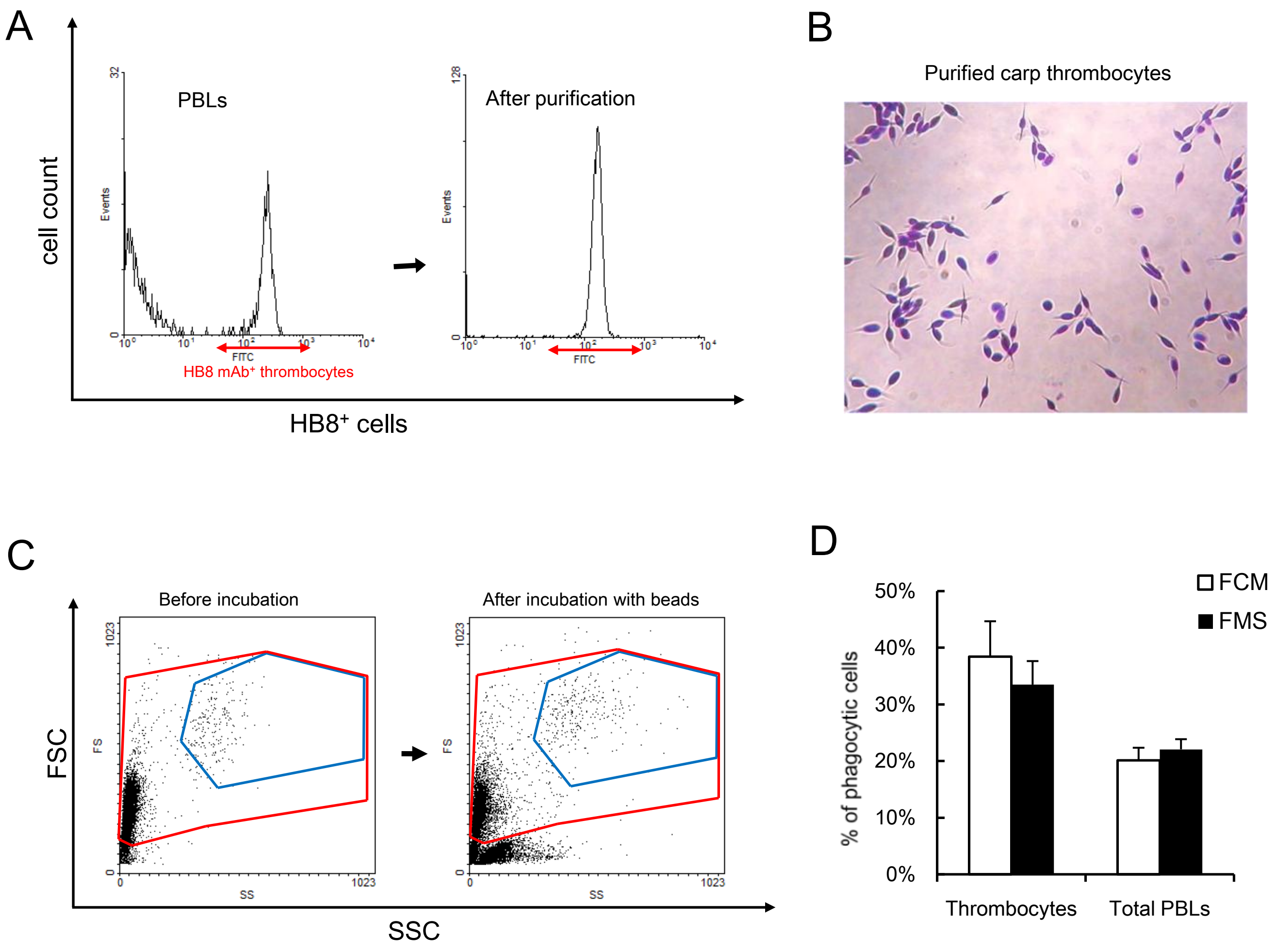

Figure S1: Purification of HB8 mAb⁺ thrombocytes. (A) Flow cytometry of carp PBLs (left) and purified thrombocytes (right) stained with HB8 mAb. (B) The morphology of purified HB8⁺ cells stained with Giemsa stain solution. Original magnification, x200. Data is representative of five independent experiments. (C) Flow cytometry scattergram of carp PBLs before (left) and after (right) incubation with latex beads (1 μm in diameter). Granulocytes were gated for the measurement of phagocytic activity. A representative result of five independent experiments is shown. Abbreviations: FSC, forward scatter; SSC, side scatter. (D) Phagocytic activities of carp thrombocytes measured by flow cytometry and fluorescent microscopy. In the microscopy, at least 300 cells were counted in each fish and percentages of phagocytic cells were calculated. The percentages of ingesting cells are shown as mean + SD determined from five independent experiments. Abbreviations: FCM, flow cytometry; FMS, fluorescent microscopy.

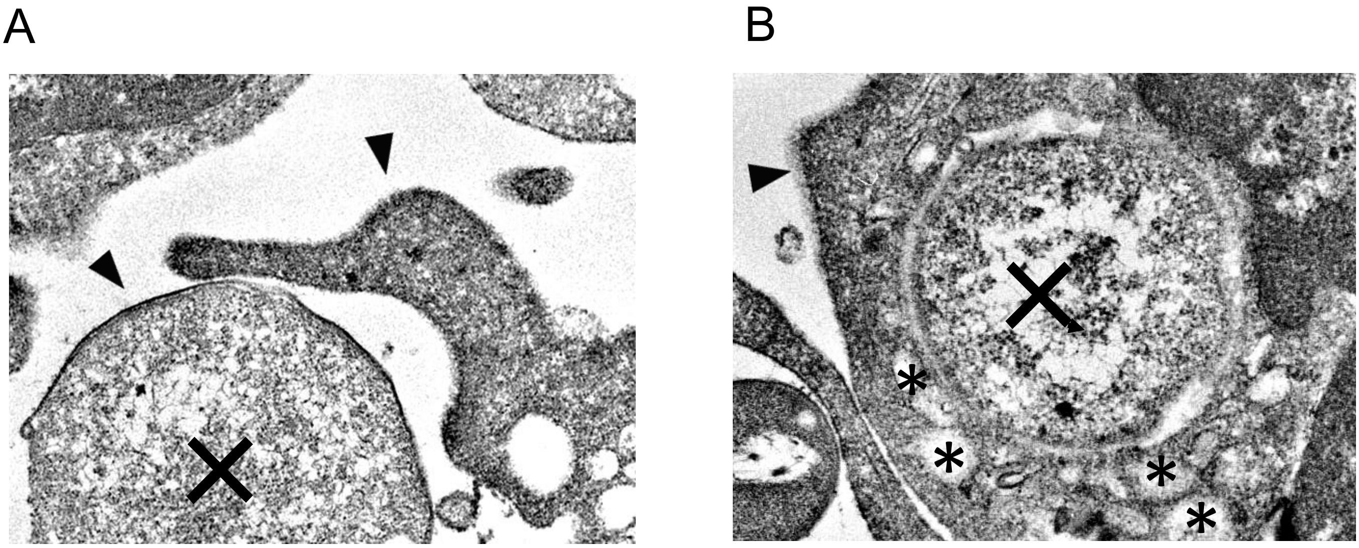

Figure S2. Detection of extracellular surfaces and ultrastructure of phagosome-like vesicles. Cell surfaces of thrombocytes and extracellular bacteria (A and B, arrowheads) were stained with tannic acid. Intracellular bacteria (B) were not stained with tannic acid. Small vesicles surrounded ingested bacteria (B, asterisks). X, bacteria. Original magnification, x10,000. Data are representative of three independent experiments.
