# Supplementary material for: Phagocytosis by Thrombocytes is a Conserved Innate Immune Mechanism in Lower Vertebrates
Source: Front Immunol. 2014 Sep 16;5:445. doi: 10.3389/fimmu.2014.00445 (PMC4165319; doi:10.3389/fimmu.2014.00445)
Supplement: Supplementary file 2 [file Data_Sheet1.DOCX]

**Supplementary Table 1.** Primers used for RT-PCR.

|  |  |  |  |  |
| --- | --- | --- | --- | --- |
|  | Primer sequence (5' - 3') | Product size (bp) | cycles | annealing temp. (ºC) |
| CD41b (AB429305) | CGTCAACACAACCAATCCTG | 198 | 30 | 56 |
|  | TGACTGAGCCGTTCTCCTTT |  |  |  |
|  |  |  |  |  |
| IgM H chain (AB004108) | ATGGAACAGCAACCTTCATGTGTTT | 228 | 30 | 55 |
|  | CTGTCTTGTGCACGAACTCACACTT |  |  |  |
|  |  |  |  |  |
| TCRα (EU025122) | CAGTGCGATATTCAGGCAAA | 243 | 34 | 56 |
|  | CGAGGATTTTCATGACACGTT |  |  |  |
|  |  |  |  |  |
| mpx (AB429306) | ACCACAGTATACCAGGCTATAATGC | 218 | 34 | 56 |
|  | GATGAGGCAGGCAAAAAGAG |  |  |  |
|  |  |  |  |  |
| IL-1β (AJ245635) | ACCAGCTGGATTTGTCAGAAG | 465 | 28 | 52 |
|  | ACATACTGAATTGAACTTTG |  |  |  |
|  |  |  |  |  |
| lysozyme (AB084624) | CAATGCTTTAAAGGGGGAATAGCAG | 156 | 28 | 56 |
|  | TGCAGTTCTTCAGTAACCCTTGCTT |  |  |  |
|  | Primer sequence (5' - 3') | Product size (bp) | cycles | annealing temp. (ºC) |
| inducible NO synthase | AGTCAGGTACTGCGTGTTTGGTCTC | 203 | 34 | 56 |
| (AJ242906) | TGTTAAACTCCTTGCATGCATCCTT |  |  |  |
|  |  |  |  |  |
| CD11-1 (AB048536) | GAGGATTCTTTGATCAGAGCAGTGT | 278 | 32 | 56 |
|  | CTTCAGATCGGCCACACAGCTGTTG |  |  |  |
|  |  |  |  |  |
| CD11-2 (AB048537) | GAGGATTCTTCGATCCGATGGATTC | 278 | 34 | 56 |
|  | CAGCTTCAGATCAGCCACACAGCTA |  |  |  |
|  |  |  |  |  |
| Integrin β2 chain | GGTGGAGAAAGTCCCAGTGA | 219 | 35 | 56 |
| (CD18-1; AB013074) | ATTTCCCCCACAGTGAGGAT |  |  |  |
|  |  |  |  |  |
| CD18 type 2 (AB031070) | TGAAAGCACAATGCCAGAAG | 254 | 32 | 56 |
|  | ATCCTCATCGGATTTTGCAC |  |  |  |
|  |  |  |  |  |
| MHC classII β DXA (X95433) | ATTTACAGCTGCACAGTGTACCACA | 189 | 28 | 56 |
|  | GATCTGTCAGTTGCAGTTGTTTCCT |  |  |  |
|  |  |  |  |  |
| β-actin (M24113) | CACTGTGCCCATCTACGAG | 221 | 24 | 56 |
|  | AGGAGGAGGAAGCAGCAGTGCCCAT |  |  |  |
|  |  |  |  |  |
